# New Contributions to Deepen the Quality-Based Safety Assessment in the Consumption of Edible Nasturtium Flowers—The Role of Volatilome

**DOI:** 10.3390/life15071053

**Published:** 2025-06-30

**Authors:** Rosa Perestrelo, Maria da Graça Lopes, Alda Pereira da Silva, Maria do Céu Costa, José S. Câmara

**Affiliations:** 1 CQM—Centro de Química da Madeira, Universidade da Madeira, Campus da Penteada, 9020-105 Funchal, Portugal; rmp@staff.uma.pt; 2 CBIOS—Research Center for Biosciences and Health Technologies, Universidade Lusófona, Av. Campo Grande, 376, 1749-024 Lisbon, Portugal; mglopes@gmail.com (M.d.G.L.); p1658@ulusofona.pt (M.d.C.C.); 3 Institute for Preventive Medicine and Public Health, Lisbon School of Medicine, University of Lisbon, 1349-017 Lisbon, Portugal; aldapsilva@medicina.ulisboa.pt; 4 Clinic of General and Family Medicine, Ecogenetics and Human Health Unity, Institute for Environmental Health, ISAMB, 1649-026 Lisbon, Portugal; 5 NICiTeS—Research Center for Health Sciences and Technologies, Polytechnic Institute of Lusophony, ERISA—Escola Superior de Saúde Ribeiro Sanches, 1950-396 Lisbon, Portugal; 6 Departamento de Química, Faculdade de Ciências Exatas e Engenharia, Universidade da Madeira, Campus da Penteada, 9020-105 Funchal, Portugal

**Keywords:** *Tropaeolum majus* L., flower and juice, headspace solid-phase microextraction, gas chromatography–mass spectrometry, volatile organic metabolites

## Abstract

The garden Nasturtium (*Tropaeolum majus* L.) is increasingly consumed worldwide due to its culinary appeal and perceived health benefits. However, the chemical markers underlying its functional properties remain insufficiently characterized. Building on evidence from a recent human pilot study confirming both high acceptability and dietary safety, we conducted a comprehensive volatilomic and phytochemical analysis of *T. majus* flowers and their juice. Headspace solid-phase microextraction coupled with gas chromatography–mass spectrometry (HS-SPME/GC-MS) was employed to establish the volatilomic fingerprint of floral tissues and juice. Our analysis revealed a striking dominance of benzyl isothiocyanate and benzonitrile, which together accounted for 88% of the total volatile organic metabolites (VOMs) in the juice, 67% and 21%, respectively. In the floral tissues, benzyl isothiocyanate was even more prevalent, representing 95% of the total volatile profile. Complementary in vitro assays confirmed a substantial total phenolic content and strong antioxidant activity in the flowers. These findings provide a robust chemical rationale for the potential health-promoting attributes of *T. majus*, while identifying key volatilomic markers that could support future functional and safety claims. In parallel, a benefit–risk assessment framework is discussed in accordance with the European Food Safety Authority (EFSA) guidelines for the Qualified Presumption of Safety (QPS) of edible flowers. Given that both benzyl isothiocyanate and benzonitrile are classified as Cramer Class III substances, a conservative intake threshold of 1.5 μg/kg body weight per day is proposed. To enable quantitative exposure modeling and support the derivation of a tolerable daily intake (TDI), future studies should integrate organic solvent-based extraction methodologies to estimate the total volatile load per gram of floral biomass. This would align risk–benefit assessments with the EFSA’s evolving framework for novel foods and functional ingredients.

## 1. Introduction

*Tropaeolum majus* L., commonly known as garden Nasturtium, is a vibrant flowering plant native to South America and widely cultivated for its ornamental appeal and culinary applications. Beyond its aesthetic and gastronomic value, *T. majus* has garnered attention for its rich phytochemical composition, encompassing vitamins, minerals, and bioactive compounds with potential health-promoting properties [1]. Recent studies have highlighted the presence of glucosinolates in *T. majus*, which upon hydrolysis yield isothiocyanates—compounds known for their antimicrobial, anti-inflammatory, and anticancer activities. Among these, benzyl isothiocyanate (BITC) has been identified as a predominant volatile organic metabolite in *T. majus* flowers and juice. BITC has demonstrated the ability to induce apoptosis and autophagy in various cancer cell lines, including prostate and breast cancer cells, through mechanisms involving reactive oxygen species generation and the modulation of signaling pathways [2]. Lin et al. [3] provided evidence that BITC effectively suppressed the growth of cultured human prostate cancer cells, causing prostate cancer cell death in a way dependent on the ROS status, and clarified the mechanism underlying BITC-induced cell death, which involves the induction of ROS production, autophagy, and apoptosis, and the relationship between these three important processes. In addition to BITC, benzonitrile (BN) is another significant volatile compound found in *T. majus*. While BN contributes to the plant’s aroma profile, its presence raises safety considerations due to its potential cytotoxic and genotoxic effects observed in vitro. Kupke et al. [4] showed by GC-MS monitored experiments that broccoli glucosinolates mainly degrade to nitriles as breakdown products, and based on the in vitro data presented, concerning genotoxicity data, concluded that it cannot be ruled out that nitriles pose a risk under conditions relevant for food consumption. Therefore, comprehensive profiling of these compounds is essential to assess the health benefits and risks associated with *T. majus* consumption.

Despite the growing interest in edible flowers as functional foods, there remains a paucity of data regarding the detailed chemical characterization and safety evaluation of *T. majus*. This study aims to fill this gap by conducting an in-depth volatilomic and phytochemical analysis of *T. majus* flowers and juice, utilizing headspace solid-phase microextraction coupled with gas chromatography–mass spectrometry (HS-SPME/GC-MS). Furthermore, we assess the antioxidant activity and total phenolic content to elucidate the potential health benefits. In alignment with the European Food Safety Authority (EFSA) guidelines, a contribution to a further benefit–risk assessment is envisaged, considering the Threshold of Toxicological Concern (TTC) for compounds of interest, to inform the safe incorporation of *T. majus* into functional foods and nutraceuticals. If the estimated exposure to a substance is higher than the relevant TTC value, a non-TTC approach is required to conclude potential adverse health effects, a task for expert groups in the regulatory authorities [5]. With this study, we envisage setting the stage for *T. majus* within the broader landscape of so-called “functional foods”, bearing a function claim [6], highlighting the significance of its bioactive compounds, and underscoring the necessity for comprehensive chemical and safety evaluations. This work aims to deepen knowledge of the volatilomic and phytochemical composition of *T. majus* L. floral tissues and their derived juice, focusing on identifying the key bioactive markers and evaluating their relevance for functional claims and nutrient applications. This investigation is driven by increasing interest in edible flowers as sources of bioactive compounds, particularly glucosinolate-derived volatiles such as isothiocyanates, which are associated with diverse biological activities. However, limited studies have characterized the volatile organic metabolite (VOM) profile of *T. majus* in both its fresh and processed forms, or evaluated the chemical shifts induced by typical culinary processing methods. Furthermore, a lack of standardization in analytical approaches has hindered the establishment of the chemical fingerprints necessary for regulatory evaluation and functional claim development. By applying advanced analytical techniques and chemometric tools, this study seeks to address these gaps and provide a robust foundation for the future exploration of *T. majus* as a functional ingredient aligned with food safety and quality frameworks, including those defined by the European Food Safety Authority (EFSA).

## 2. Materials and Methods

### 2.1. Chemicals and Reagents

Analytical-grade chemicals and reagents were all utilized. Methanol of HPLC quality was acquired from Fischer Scientific in Loughborough, UK. Sigma-Aldrich (St. Louis, MO, USA) provided the following: gallic acid (purity > 99%), quercetin (≥98%), sodium chloride (NaCl, 99.5%), 3-octanol (internal standard, 99%), 6-hydroxy-2,5,7,8-tetramethylchromane-2-carboxylic acid (Trolox, 98.0%), and anhydrous sodium carbonate (Na_2_CO_3_, 99.8%). Purchased from Riedel-de Haën^®^ (Seelze, Germany) was aluminum chloride (AlCl_3_). The following were provided by Fluka (Buchs, Switzerland): potassium persulfate (99.0%), 1,1-diphenyl-2-picrylhydrazyl (DPPH·≈ 90%) in its free radical form, 2,2’-azinobis-(3-ethylbenzothiazoline-6-sulfonic acid) radical cation (ABTS, 98.0%), and an alkane series (C_8_ to C_20_, 40 mg/L in n-hexane). Supelco (Bellefonte, PA, USA) supplied the glass vials, SPME holder for manual sampling, and the divinylbenzene/carboxen/polydimethylsiloxane (DVB/CAR/PDMS) coated SPME fiber (50/30 µm, 1 cm). Ultrapure water (H_2_O, 18 MΩ cm) was provided by the Milli-Q water purification system (Millipore, Milford, MA, USA).

### 2.2. Samples and Sample Treatment

**Figure 1:**
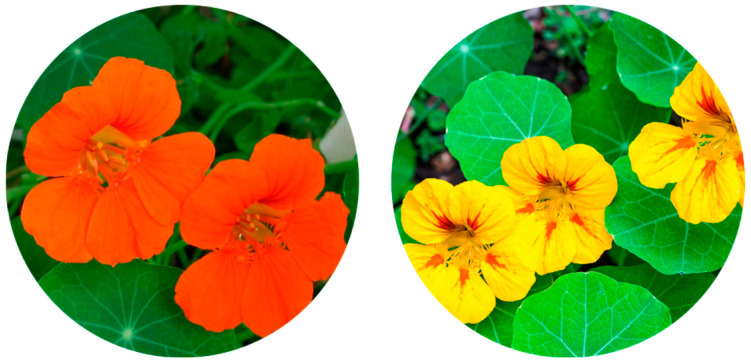
Image of the analyzed samples of *Tropaeolum majus* L.

*Tropaeolum majus* L. flowers (Figure 1) of spontaneous growth were formerly freshly harvested in the early morning around Vila Franca (Portugal), at Latitude 132 38.955834, Longitude −8.994359, from Dr Luis César Pereira Urban Park, July 2019, Time: 2.40 p.m. The first manual harvest was carried out by Maria da Graça Patrício Serrador, and a specimen was deposited in the Herbarium LISU-Botanical Garden (Voucher no. LISU270425, 2019) [7]. *Tropaeolum majus* L. flowers for the present study were again harvested in the early morning on 12 July 2023, at Latitude 38.955834, Longitude −8.994359, Dr. Luis César Pereira Urban Park, Time: 9:40 a.m., to minimize diurnal variation in metabolite composition, and the plant material was immediately selected for uniformity and integrity. Within 30 min of collection, samples were placed in airtight, vacuum-sealed polyethylene bags and transferred to a −20 °C freezer to preserve their biochemical composition. Samples were stored under frozen conditions for a maximum of 20 days before analysis. To minimize loss or transformation of volatile compounds, samples were not subjected to any thawing before analysis. For volatilomic analysis, frozen flower tissues were finely ground under cooled conditions and promptly subjected to HS-SPME/GC-MS.

### 2.3. Total Phenolic Content

The Folin–Ciocalteu method, which was modified from Figueira et al. [8], was applied to calculate the total phenolic content (TPC) of Nasturtium flower extract. In short, 300 µL of extract was mixed with 1.5 mL of Folin–Ciocalteu solution (1:10 *v*/*v*) and 1.2 mL of 7.5% (*w*/*v*) Na_2_CO_3_ solution. After homogenizing the mixture, it was incubated at 25 ± 1 °C for 30 min in the dark. A UV-Vis spectrophotometer (Lambda 25, Perkin Elmer, Waltham, MA, USA) was used to register the absorbance at 765 nm following the incubation. A calibration curve (R^2^ = 0.992) made using gallic acid standard (15 to 76 mg/L) was used to determine the TPC, which was represented in mg of gallic acid equivalent (GAE)/100 g of dry weight (DW).

### 2.4. Determination of Antioxidant Properties from Nasturtium Flower Extract

#### 2.4.1. DPPH Assay

With minor modifications, the 2,2-diphenyl-1-picrylhydrazyl (DPPH) test was performed in accordance with Figueira et al. [8] to determine the Nasturtium flower extract’s capacity to scavenge free radicals. Absorbance was determined at 515 nm after 100 µL of the extract reacted with 3.9 mL of DPPH working solution (absorbance ~0.900 ± 0.01) for 45 min at 25 ± 1 °C in the dark. A calibration curve (25–600 µg/mL Trolox, R^2^ = 0.989) was used to determine the antioxidant properties, which were evaluated in triplicate and represented as mg Trolox equivalent (TE)/100 g dry weight (DW).

#### 2.4.2. ABTS Assay

A modified version of the Figueira et al. [8] protocol was used to measure the antioxidant activity of Nasturtium flower extract against the stable ABTS^+^ radical cation using 2,2′-azino-bis(3-ethylbenzothiazoline-6-sulfonic) acid (ABTS). After reacting ABTS (20 mM) with potassium persulfate (70 mM) and letting it sit in the dark for 16 h, ABTS·^+^ radical cations were produced. PBS was used to dilute the ABTS solution until an absorbance value of around 0.900 ± 0.01 was achieved. Then, 3 mL of the diluted ABTS solution was mixed with 12 μL of the extract. Absorbance was measured at 734 nm, and antioxidant activity was expressed as mg Trolox equivalent (TE)/100 g dry weight using a calibration curve (100–1500 µg/mL Trolox, R^2^ = 0.997), with all measurements performed in triplicate.

### 2.5. HS-SPME Extraction

The HS-SPME process used in the current study was adopted from Izcara et al. [9], with slight modifications. Following the manufacturer’s instructions, the SPME fiber was thermally conditioned for 10 min each day before usage to confirm there were no carryover analytes. A 10 mL amber glass vial with a stirring bar (2 × 0.5 mm, 450 rpm) was filled with 1 g of sample, 5 mL of H_2_O, 10 μL of 3-octanol (100 μg/mL), and 0.5 g of NaCl to extract VOMs from Nasturtium flower and juice. A PTFE-faced silicone septum was used to seal the vial, and the vial was placed in a thermostatic block at 45 ± 1 °C. The SPME fiber (DVB/CAR/PDMS) was inserted into the headspace sample vial for 50 min to absorb VOMs in the fiber. Finally, to thermally desorb the target analytes, the fiber was withdrawn into the holder needle, taken out of the vial, and put into the GC-MS injector at 250 °C for 6 min. All analyses were carried out in triplicate (n = 3).

### 2.6. GC-qMS Conditions

An Agilent Technologies 6890 N (Palo Alto, CA, USA) gas chromatography system fitted with a SUPELCOWAX^®^ 10 fused silica capillary column (60 m × 0.25 mm i.d. × 0.25 μm film thickness) supplied by Supelco (Bellefonte, PA, USA) was used to perform chromatographic separations of the VOMs from Nasturtium flowers and juice. The carrier gas was helium (Helium N60, Air Liquid, Portugal) with a flow rate of 1 mL/min and a column-head pressure of 13 psi. The following was the temperature program: 40 °C for 1 min, 220 °C with a ramp of 2.5 °C/min, and then kept isothermally at 220 °C for 10 min. MS detection was performed by an Agilent 5975 quadrupole inert mass selective detector in full scan mode. The injector and the quadrupole mass spectrometry detector had respective temperatures of 250 °C and 230 °C. A 0.75 mm i.d. insert was used in conjunction with a splitless injector to conduct the injection. The ion energy for electron impact ionization (EI) was 70 eV. The obtained mass range was between 30 and 300 *m*/*z*. By comparing the RTs, KI, and mass spectra with standards, and the mass spectra with the National Institute of Standards and Technology’s (NIST) MS 05 spectral database (Gaithersburg, MD, USA), the VOMs were inferred. The van Den Dool and Kratz (1963) equation [10] was used to determine the KI values. The KI was calculated using an n-alkane solution from the C8 to C20 series, and the results were compared, where available, to those found in online databases (Pherobase, Flavornet, PubChem) and the literature for comparable columns [11,12,13]. The relative concentration of each VOC was determined by multiplying its relative peak area (VOM peak area/ IS peak area) by the amount of 3-octanol added, and expressed as µg/kg of 3-octanol equivalent.

### 2.7. Statistical Analysis

The statistical analysis of the data was conducted using the MetaboAnalyst 6.0 web-based tool [14]. After removing VOMs with missing values by pre-processing, the raw GC-qMS data were normalized (data transformation using the cubic root and data scaling using autoscaling). To find significant differences, the dataset was then put through a *t*-test of Nasturtium flowers and juice at *p* < 0.05. Partial least squares-discriminant analysis (PLS-DA) and principal component analysis (PCA) were employed to identify VOMs that would indicate variations across the sample sets and shed light on the separations between the Nasturtium flowers and juice under investigation. Potential candidates for characterizing the research samples were VOMs differentially expressed in the univariate analysis and having a variable importance in the projection (VIP) score greater than 1. Using the 15 most significant with VIP > 1, as produced by Ward’s algorithm and Euclidean distance analysis, hierarchical cluster analysis (HCA) was performed to identify clustering patterns for the characterization of the Nasturtium flowers and juice under examination.

## 3. Results and Discussion

### 3.1. Volatilomic Fingerprint of Nasturtium

**Figure 2:**
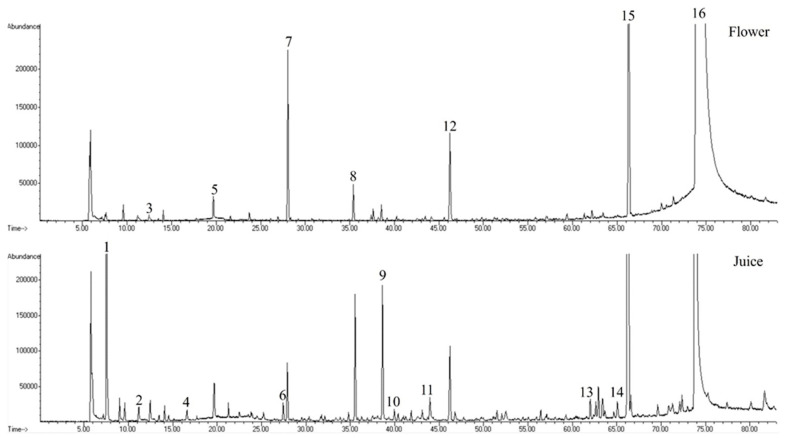
Typical GC-MS profile of Nasturtium flowers and juice. Peak identification: (1) acetaldehyde, (2) ethyl acetate, (3) 2-ethyl furan, (4) 1-penten-3-one, (5) hexanal, (6) 2-pentyl furan, (7) (*Z*)-2-hexenal, (8) 1-hexanol, (9) (*E*)-2-hexen-1-ol, (10) ethyl octanoate, (11) decanal, (12) benzaldehyde, (13) geranylacetone, (14) phenylethyl alcohol, (15) benzonitrile, (16) benzyl isothiocyanate.

Chromatograms for Nasturtium flowers and juice were obtained following the HS-SPME extraction and GC-MS analysis (Figure 2).

Following the interpretation of these chromatograms, a total of 78 VOMs were identified, including 22 aldehydes, 13 esters, 11 alcohols, 7 terpenoids, 7 hydrocarbons, 6 ketones, 3 furans, 2 isothiocyanates, and others. All of the VOMs found in Nasturtium flowers and juice are shown in Table 1, along with their peak area and relative peak area.

**Figure 3:**
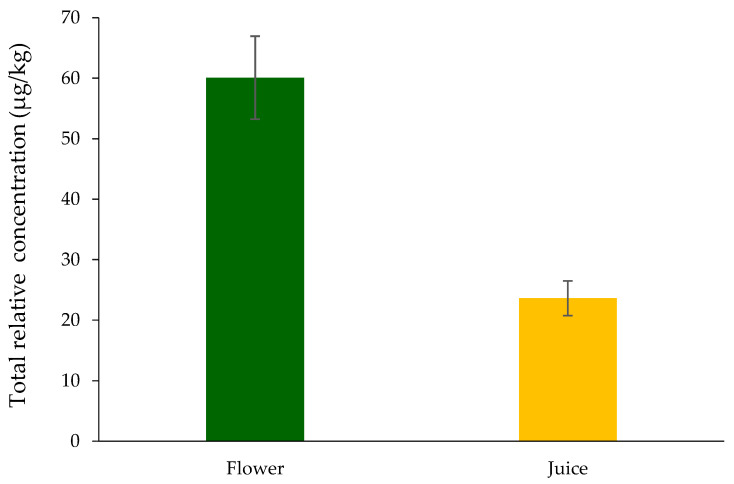
Relative concentration (µg/kg) of identified VOMs in Nasturtium flowers and juice.

As can be observed in Table 1, the volatilomic profile of Nasturtium varied extensively between the flower and its corresponding juice. The juice possessed a much broader and intense volatilomic profile with greater numbers of compounds identified and lower total peak areas (Figure 3). Juicing damages plant tissue mechanically, which initiates enzymatic activity (lipoxygenase, myrosinase, among others) and facilitates the release or generation of VOMs through lipid peroxidation, glycoside cleavage, and other metabolic processes.

**Figure 4:**
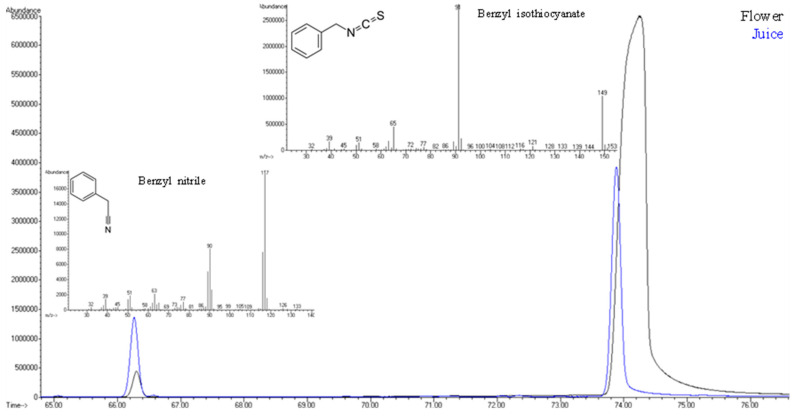
Overlap of the two main identified VOMs in Nasturtium flowers and juice—benzonitrile and benzyl isothiocyanate.

When compared to Nasturtium flowers, aldehydes such as hexanal, (*E*)-2-pentenal, nonanal, and decanal have significantly highest peak areas in the juice, indicating greater lipid oxidation or amino acid breakdown during juice processing. Moreover, aldehydes such as 2-methylpropanal, (*E*)-2-heptenal, and (*E*)-2-octenal were only found in the Nasturtium juice (Table 1). The juice also contains much higher content of alcohols such as 1-hexanol, (*E*)-2-hexen-1-ol, and phenylethyl alcohol, which is in line with studies that alcohol levels rise during tissue rupture and fermentation-like activity. The juice primarily contained esters (e.g., ethyl acetate, ethyl octanoate, methyl benzoate) and ketones (e.g., 3-pentanone, 1-penten-3-one, 1-decen-3-one), suggesting that processing conditions favor their synthesis or release. On the other hand, the flowers exhibit an extremely high peak area for a nitrile chemical family (21.3% of total volatilomic fraction) and a lower peak area for isothiocyanate (67.1%) compared to flowers, where nitrile and isothiocyanate represent 2.2% and 94.6% of total volatilomic fraction, respectively. Figure 4 shows the overlapping of these two VOMs identified in Nasturtium flowers and juice, which is responsible for the highest contribution of nitriles and isothiocyanates to the total volatilomic fraction.

Similar to the high levels of benzyl isothiocyanate observed here, prior work has reported glucosinolate-derived VOMs as major aroma contributors in *Tropaeolum* and other glucosinolate-rich species [9,15,16].

### 3.2. Odor of Some Identified VOMs and Their Potential Bioactive Effects

Table 2 shows the most abundant VOMs identified in Nasturtium flowers and juice, as well as the respective odor of each VOM [11,12] and the beneficial potential effects reported in the literature [17,18,19,20,21,22].

The VOMs identified in Nasturtium flowers and juice contribute significantly to the overall aroma profile, with predominant odor descriptors such as fruity, citrus, green, floral, and sweet. These sensory properties are crucial for interactions between the plants and their surroundings, such as drawing pollinators and discouraging herbivores. The most common biological activity of the VOMs, aside from their aroma, is antibacterial activity. This implies use in natural food preservation and a protective function in plant physiology. Furthermore, some VOMs, such as limonene, β-myrcene, and benzyl isothiocyanate, have other bioactivities, such as chemoprotective, anti-inflammatory, antioxidant, and anticancer activities. Moreover, several studies have demonstrated that the predominant VOM identified in both samples, benzyl isothiocyanate, showed anticancer properties by inhibiting the genesis, development, and metastasis of human tumors [23]. In the same way, benzyl isothiocyanate, which was reported to demonstrate a strong bactericidal activity against oral pathogens and other Gram-negative bacteria, was shown by Sofrata et al. [24]. These findings highlight the VOMs’ dual ecological and therapeutic potential and call for further research on their beneficial uses in industrial and medical settings. The phytochemical composition and antioxidant activity of *T. majus* were assessed through TPC and antioxidant assays (ABTS and DPPH). The sample exhibited a TPC of 2.6 ± 0.5 mg GAE/100 g, indicative of a moderate phenolic profile when compared to other edible flowers [25]. Regarding antioxidant capacity, the ABTS assay yielded a relatively high value of 169 ± 19 mgTE/100 g DW, reflecting a broad-spectrum radical scavenging ability. Conversely, the DPPH value was 19 ± 1 mg TE/100 g DW, which is consistent with the expected lower reactivity of DPPH toward certain antioxidant compounds. These results support the potential of *T. majus* as a source of bioactive compounds, although further comparisons with other edible flowers and plant matrices would help contextualize its nutraceutical relevance.

### 3.3. Bridging Qualitative Findings with Potential Health Risks

Benzyl isothiocyanate (BITC) and benzonitrile (BN) are naturally occurring compounds found in cruciferous and glucosinolate-containing plants such as *T. majus*. While both have been explored for bioactive potential, including antimicrobial and chemopreventive effects, they also pose health risks that warrant careful toxicological evaluation. BITC has been shown to induce oxidative stress, genotoxicity, and cytotoxicity in various in vitro and in vivo models. For instance, it can cause DNA strand breaks and apoptosis in human liver and cancer cells at low micromolar concentrations, possibly due to its electrophilic nature and capacity to generate reactive oxygen species (ROS) [26,27]. In rodents, sub-chronic exposure has been associated with liver and kidney toxicity and altered lipid metabolism, prompting concerns about cumulative effects from dietary sources.

BN is a substance of concern primarily because of its metabolic conversion to cyanide, a potent mitochondrial toxin. BN itself exhibits low acute toxicity in animal studies but can produce adverse effects upon biotransformation, particularly under conditions that promote cyanide release [28]. In vitro assays also report that BN can induce DNA damage in human hepatic cells, suggesting potential genotoxic risk [27,29]. Both compounds are classified in Cramer Class III, indicating a higher level of toxicological concern based on structure–activity relationships. Thus, for functional foods or supplements containing high proportions of BITC and BN, such as nasturtium juice, regulatory agencies such as the EFSA recommend applying conservative exposure thresholds, such as the Threshold of Toxicological Concern (TTC) of 1.5 µg/kg bw/day, until more specific toxicokinetic and human safety data become available.

To address the derivation of a tolerable daily intake (TDI) for the BITC and BN present in nasturtium juice, it is essential to consider both the data requirements for a robust risk assessment and the limitations inherent in defining a Benchmark Dose (BMD) [30]. A first step should be a quantitative analysis of VOMs, employing solvent-based extraction methods to quantify the actual concentrations of BITC and BN in the edible parts of *T. majus*. This addresses the limitation of HS-SPME/GC-MS, which, while effective for profiling, does not provide absolute quantification. Then, comprehensive toxicological evaluations, including acute, sub-chronic, and chronic toxicity studies in relevant animal models, are desirable. These studies should identify critical endpoints and dose-response relationships for BITC and BN, along with pharmacokinetic data [31,32]. Additionally, estimating human exposure levels is mandatory, based on consumption patterns of nasturtium products, considering different population groups and dietary habits. The possibility of application of the BMD for dose-response modeling is preferred over the traditional NOAEL method due to its statistical robustness and ability to utilize all available data points. The BMD approach involves identifying a dose that produces a predefined change in response rate (the benchmark response) and calculating its lower confidence limit (BMDL) to serve as the reference point for risk assessment according to the most recent EFSA Guidance for foods [33].

By addressing these data requirements and acknowledging the limitations of the BMD approach, future research can enhance the risk assessment of BITC and BN in nasturtium, ultimately supporting the establishment of a TDI that ensures consumer safety.

### 3.4. Multivariate Statistical Analysis

**Figure 5:**
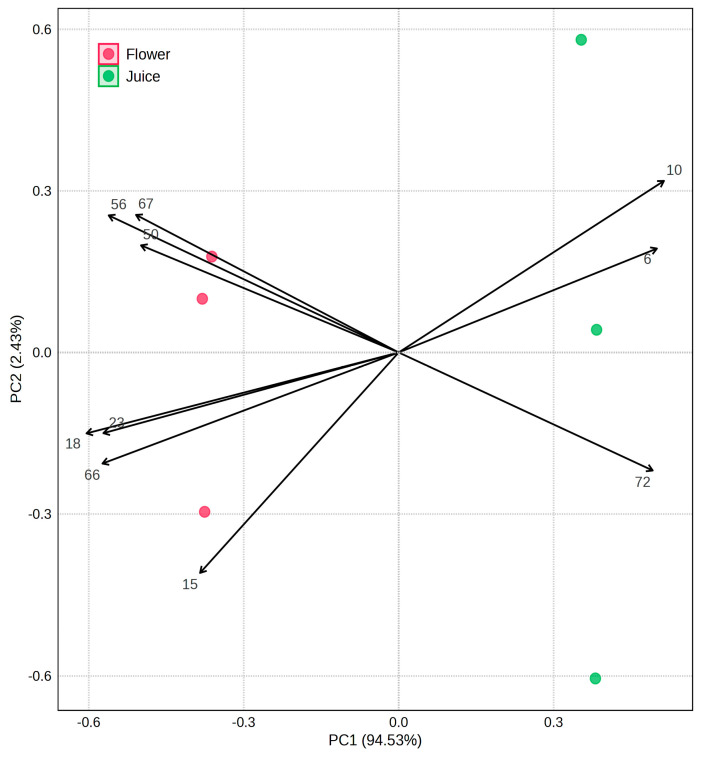
PCA biplot of the VOMs identified in the investigated Nasturtium flowers and juice. The numbers correspond to the VOMs (see Table 1).

A multivariate statistical analysis was performed by the MetaboAnalyst 6.0 program to obtain the principal component analysis (PCA) and partial least squares-discriminant analysis (PLS-DA), as described in Section 2.7. According to the *t*-test results, the VOMs with *p* ≥ 0.05 indicate no statistically significant difference between the flower and juice samples, namely ethyl acetate (6), toluene (10), limonene (15), ethyl dodecanoate (50), hexanoic acid (67), and methyl tetradecanoate (72). Figure 5 shows the PCA biplot, which allows visualization of the clear differentiation between the flowers and juice. The variance of the first principal component (PC1) was 94.5%, while the second (PC2) was 2.4%, so the total of both components was 96.9% of the data variability.

**Figure 6:**
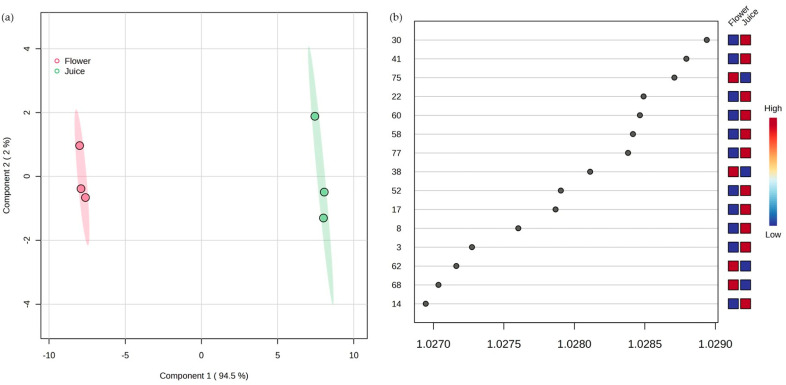
Statistical analysis using PLS-DA score plot (**a**) and variable importance in the projection (VIPs) plot (**b**) of the VOMs identified in the analyzed Nasturtium flowers and juice. The numbers correspond to the VOMs (see Table 1).

As shown in Figure 6a, the PLS-DA score plot shows unique volatilomic profiles and a clear distinction between flower and juice samples along the first component (Component 1, 94.5%). Key discriminant VOMs, including compounds (*Z*)-3-hexen-1-ol (30), 2-ethyl-1-hexanol (41), and styrene (22), are identified in the corresponding VIP score plot (Figure 6b), these VOMs being important contributors to the separation.

**Figure 7:**
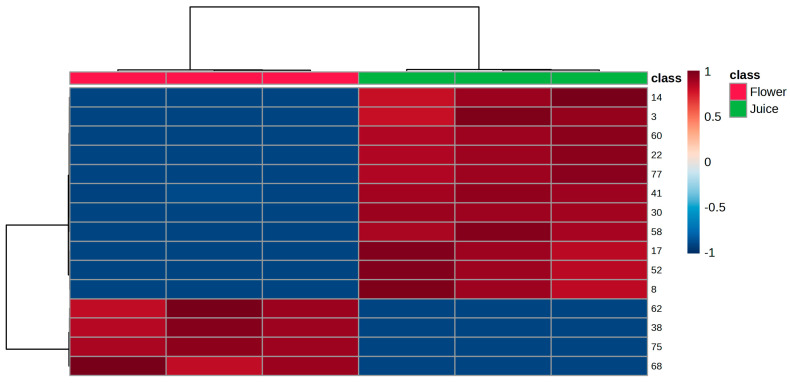
HCA using the 15 significant VOMs identified in the analyzed Nasturtium flowers and juice, as obtained by the *t*-test. The color of the graphic ranges from dark red, which indicates high correlation of VOMs with the corresponding samples, to light blue, which indicates low association of VOMs with the target.

The PLS-DA of the VOMs with VIP values > 1 confirms each sample’s unique volatilomic fingerprint and demonstrates a strong distinction between the juice and flower samples. As can be observed in Figure 7, the heatmap shows two main clusters: one of compounds with consistently greater abundance in juices, namely 4-methyl-3-heptanone (14), 2-methylpropanal (3), 1,2-dimethoxy benzene (60), methyl hexadecanoate (77), 2-ethyl-1-hexanol (41), (*Z*)-3-hexen-1-ol (30), dodecanal (58), 2-pentyl furan (17), menthol (52) and 3-pentanone (8), and the other with VOMs having relatively greater or variable abundance in flowers, namely naphthalene (62), dimethyl styrene (38), 3-phenyl-2-propenal (75), and geranylacetone (68). This tendency for clustering also supports the discriminating ability of the selected VOMs in differentiating the two groups of samples.

## 4. Conclusions

This study establishes the first comprehensive volatilomic profile of *Tropaeolum majus* L. (nasturtium) floral tissue and its processed juice through advanced HS-SPME/GC-MS analysis, revealing statistically significant disparities (*p* < 0.05) in VOMs between matrices. Key findings demonstrate volatilomic divergence and marker identification. The floral tissue exhibited 48 unique VOMs with twice the total peak area intensity of juice (59 VOMs), indicating substantial biochemical modifications during processing. Benzyl isothiocyanate dominated both matrices (95% floral vs. 67% juice), followed by benzonitrile (21% juice), suggesting enzymatic modulation during juice extraction. Multivariate analysis (PCA, PLS-DA, HCA) identified 15 VOMs as differentiation markers, namely 4-methyl-3-heptanone (14), 2-methylpropanal (3), 1,2-dimethoxy benzene (60), methyl hexadecanoate (77), 2-ethyl-1-hexanol (41), (*Z*)-3-hexen-1-ol (30), dodecanal (58), 2-pentyl furan (17), menthol (52) and 3-pentanone (8), 3-phenyl-2-propenal (75), and geranylacetone (68). The prevalence of benzyl isothiocyanate—a potent chemopreventive agent with demonstrated antimicrobial and antioxidant capacities—positions nasturtium-derived products as viable candidates for natural preservatives in food systems, bioactive additives in nutrient-healthy sources targeting oxidative stress pathways, and sustainable flavor enhancers leveraging odor-active VOMs (e.g., citrus, floral). While the juice’s complex volatilome (59 VOMs) shows superior functional claim ingredient potential, critical gaps remain in vivo bioavailability of isothiocyanate metabolites, in the sensory perception thresholds of dominant VOMs in food matrices, and the toxicological risk assessment aligned with the EFSA’s scientific guidance and European parliament regulatory framework. These findings provide a chemical rationale for leveraging nasturtium in circular bioeconomy strategies, although translational success hinges on resolving pharmacokinetic and regulatory challenges through targeted preclinical studies.

## Figures and Tables

**Table 1:** Volatile organic metabolites (VOMs) identified in Nasturtium flowers and juice and their respective retention time (RT), relative concentration (µg/kg), and percentage relative peak area (%RPA), as determined by HS-SPME/GC-MS.

RT (min)	Peak nº	KI Cal ^a^	KI Lit ^b^	VOMs	Chemical Family	Relative Concentration (µg/kg) (RSD)				%RPA (RSD)			
						Flower		Juice		Flower		Juice	
7.17	1	<800	<800	Acetaldehyde	Aldehyde	-		0.011	(10)	-		0.048	(12)
7.77	2	<800	<800	Dimethyl sulfide	Sulfur	-		0.003	(18)	-		0.011	(24)
8.98	3	837	837	2-Methylpropanal	Aldehyde	-		0.005	(8)	-		0.021	(10)
9.31	4	853	850	Methyl acetate	Ester	-		0.006	(24)	-		0.026	(25)
10.32	5	900	901	Tetrahydrofuran	Furan	0.002	(11)	-		0.004	(1)	-	
10.96	6	919	921	Ethyl acetate	Ester	0.003	(23)	0.004	(7)	0.005	(33)	0.015	(14)
13.51	7	933	923	2-Ethyl furan	Furan	0.004	(13)	0.018	(15)	0.007	(22)	0.077	(3)
14.55	8	948	956	3-Pentanone	Ketone	-		0.019	(5)	-		0.083	(10)
16.6	9	998	991	1-Penten-3-one	Ketone	-		0.037	(17)	-		0.159	(18)
17.78	10	1025	1030	Toluene	H	0.007	(6)	0.009	(15)	0.011	(17)	0.037	(21)
19.68	11	1065	1067	Hexanal	Aldehyde	0.064	(6)	0.132	(20)	0.107	(6)	0.553	(8)
22.73	12	1125	1125	(*E*)-2-pentenal	Aldehyde	0.003	(3)	0.019	(15)	0.005	(8)	0.082	(10)
23.72	13	1145	1158	β-Myrcene	Terpenoid	0.024	(20)	-		0.041	(31)	-	
23.74	14	1146	1151	4-Methyl-3-heptanone	Ketone	-		0.013	(9)	-		0.054	(15)
26.11	15	1188	1198	Limonene	Terpenoid	0.007	(20)	0.005	(15)	0.012	(28)	0.020	(17)
26.94	16	1203	1204	(*Z*)-2-hexenal	Aldehyde	0.012	(2)	0.006	(7)	0.02	(13)	0.024	(11)
27.03	17	1205	1214	2-Pentyl furan	Furan	-		0.051	(5)	-		0.217	(10)
27.90	18	1222	1225	(*E*)-2-hexenal	Aldehyde	0.639	(16)	0.205	(13)	1.093	(24)	0.876	(14)
28.38	19	1232	1236	Ethyl hexanoate	Ester	0.009	(15)	-		0.016	(24)	-	
29.19	20	1247	1235	Methyl isothiocyanate	I	0.008	(17)	-		0.014	(26)	-	
29.31	21	1249	1240	3-Octanone	Ketone	-		0.011	(15)	-		0.047	(11)
29.70	22	1256	1262	Styrene	H	-		0.009	(5)	-		0.039	(12)
30.76	23	1276	1275	Hexyl acetate	Ester	0.007	(17)	0.004	(7)	0.012	(28)	0.017	(11)
31.41	24	1287	1278	Octanal	Aldehyde	-		0.012	(8)	-		0.049	(11)
32.43	25	1306	1310	1-Octen-3-one	Ketone	-		0.017	(15)	-		0.071	(16)
33.95	26	1336	1312	3-Heptanol	Alcohol	-		0.015	(15)	-		0.066	(17)
34.22	27	1342	1338	(*E*)-2-heptenal	Aldehyde	-		0.010	(11)	-		0.043	(4)
34.92	28	1355	1365	6-Methyl 5-hepten-2-one	Ketone	0.006	(21)	0.027	(12)	0.01	(28)	0.117	(14)
35.38	29	1364	1357	1-Hexanol	Alcohol	0.107	(2)	0.396	(4)	0.181	(11)	1.696	(10)
36.36	30	1383	1374	(E)-3-hexen-1-ol	Alcohol	-		0.012	(1)	-		0.053	(10)
37.41	31	1383	1389	(Z)-3-hexen-1-ol	Alcohol	0.022	(22)	-		0.039	(33)	-	
38.19	32	1419	1419	Nonanal	Aldehyde	0.018	(17)	0.142	(3)	0.031	(28)	0.610	(11)
38.60	33	1428	1420	(*E*)-2-hexen-1-ol	Alcohol	0.045	(4)	0.507	(15)	0.075	(8)	2.163	(16)
39.20	34	1441	-	4-Methyl-2,4-hexadiene	H	0.011	(19)	-		0.02	(30)	-	
40.23	35	1463	1457	Ethyl octanoate	Ester	0.014	(9)	0.028	(4)	0.023	(20)	0.120	(12)
40.35	36	1465	1467	(*E*)-2-octenal	Aldehyde	-		0.029	(18)	-		0.124	(16)
40.52	37	1469	1474	Decanal	Aldehyde	0.006	(22)	-		0.011	(32)	-	
40.98	38	1478	1462	Dimethyl styrene	H	0.008	(8)	-		0.013	(19)	-	
42.56	39	1493	1498	Acetic acid	Acid	-		0.031	(17)	0.015	(6)	-	
42.58	40	1495	1497	(*E*,*E*)-2,4-heptadienal	Aldehyde	0.009	(18)	-		-		0.133	(18)
43.12	41	1511	1503	2-Ethyl-1-hexanol	Alcohol	0.008	(4)	0.027	(3)	0.014	(15)	0.114	(10)
43.49	42	1519	1546	β-Cubebene	Terpene	0.014	(12)	-		0.024	(23)	-	
44.15	43	1524	1519	(*E*)-2-nonenal	Aldehyde	0.017	(4)	0.025	(4)	0.029	(15)	0.108	(10)
45.58	44	1532	1526	β-Bourbonene	Terpene	0.013	(14)	-		0.022	(25)	-	
46.25	45	1548	1550	Benzaldehyde	Aldehyde	0.342	(6)	0.286	(2)	0.579	(17)	1.23	(12)
46.80	46	1552	1567	1-Octanol	Alcohol	0.007	(20)	0.033	(17)	0.012	(31)	0.138	(15)
49.64	47	1567	1577	Undecanal	Aldehyde	-		0.007	(11)	-		0.031	(5)
49.83	48	1571	1581	Hexyl hexanoate	Ester	0.014	(16)	-		0.024	(27)	-	
50.32	49	1582	1573	β-Cedrene	Terpene	0.006	(13)	-		0.011	(18)	-	
51.19	50	1601	1610	Ethyl decanoate	Ester	0.012	(7)	0.009	(13)	0.02	(19)	0.038	(11)
51.26	51	1603	1602	Methyl benzoate	Ester	-		0.012	(10)	-		0.052	(5)
51.49	52	1609	1626	Menthol	Terpene	-		0.033	(4)	-		0.143	(10)
51.96	53	1621	1630	(*E*)-2-decenal	Aldehyde	-		0.026	(13)	-		0.108	(9)
52.07	54	1624	1624	1-Nonanol	Alcohol	-		0.060	(14)	-		0.254	(12)
52.12	55	1625	-	Cycloheptane	H	0.010	(16)	-		0.018	(27)	-	
52.54	56	1635	1631	Benzeneacetaldehyde	Aldehyde	0.008	(1)	0.005	(14)	0.014	(12)	0.023	(21)
53.49	57	1658	1653	Ethyl benzoate	Ester	-		0.011	(9)	-		0.045	(12)
55.03	58	1695	1695	Dodecanal	Aldehyde	-		0.013	(5)	-		0.054	(7)
55.84	59	1713	1706	2,6-Dichloroanisole	Ethers	0.019	(16)	-		0.033	(27)	-	
55.96	60	1716	1716	1,2-Dimethoxy benzene	H	-		0.012	(5)	-		0.051	(13)
57.18	61	1744	1740	1-Dodecanol	Alcohol	0.011	(12)	0.006	(10)	0.018	(21)	0.024	(9)
57.88	62	1760	1763	Naphthalene	H	0.005	10	-		0.008	(22)	-	
58.09	63	1764	1766	(*E*,*Z*)-2,4-decadienal	Aldehyde	-		0.008	(9)	-		0.036	(20)
59.39	64	1793	1798	Methyl 2-hydroxybenzoate	Ester	0.007	(13)	0.016	(1)	0.011	(25)	0.068	(10)
60.36	65	1811	1819	(*E*,*E*)-2,4-decadienal	Aldehyde	-		0.009	(8)	-		0.038	(18)
61.33	66	1837	1837	Ethyl dodecanoate	Ester	0.009	(10)	0.005	(11)	0.016	(10)	0.021	(13)
61.44	67	1840	1849	Hexanoic acid	Acid	0.015	(19)	0.009	(11)	0.025	(23)	0.038	(14)
62.17	68	1859	1859	Geranylacetone	Terpenes	0.026	(4)	-		0.044	(7)	-	
63.38	69	1890	1889	Benzyl alcohol	Alcohol	0.022	(5)	0.067	(6)	0.037	(6)	0.288	(14)
65.07	70	1907	1908	Phenylethyl alcohol	Alcohol	0.008	(14)	0.086	(18)	0.014	(25)	0.363	(13)
66.26	71	1914	-	Benzonitrile	Nitrile	1.312	(3)	5.082	(18)	2.202	(8)	21.35	(6)
68.91	72	1929	1938	Methyl tetradecanoate	Ester	0.007	(8)	0.010	(14)	0.012	(4)	0.041	(9)
69.99	73	1934	1933	Tetradecanal	Aldehyde	0.026	(13)	-		0.043	(1)	-	
70.95	74	2039	2050	Octanoic acid	Acid	-		0.062	(19)	-		0.261	(11)
71.29	75	2041	2046	3-Phenyl-2-propenal	Aldehyde	0.035	(6)	-		0.060	(17)	-	
74.06	76	2155	2143	Benzyl isothiocyanate	I	57.115	(12)	15.842	(12)	94.95	(1)	67.08	(2)
77.41	77	2171	2191	Methyl hexadecanoate	Ester	-		0.029	(5)	-		0.126	(13)
80.15	78	2284	2294	Decanoic acid	Acid	-		0.054	(16)	-		0.229	(15)

^a^; Kovats index relative n-alkanes (C8 to C20) on a SUPELCOWAX^®^ 10 capillary column; ^b^ Kovats index relative reported in the literature for equivalent capillary column [11,12,13].

**Table 2:** Odors and potential bioactive effects reported in the literature of some of the most active VOMs identified in the Nasturtium flowers and juice.

Peak nº	VOMs	Flower	Juice	Odor	Bioactive Effect
6	Ethyl acetate	x	x	Fruity, sweet, solvent	Antimicrobial
11	Hexanal	x	x	Fresh, green grass, leaf	Antimicrobial, antifungal
13	β-Myrcene	x		Citrus, fruit, wood	Analgesic, anti-inflammatory, antibiotic, anticancer, antioxidant
15	Limonene	x	x	Citrus, fruit, wood	Antimutagenic, antitumor, antioxidant, antimicrobial, antiproliferative, chemoprotective, anthelmintic, insecticidal
18	(*E*)-2-hexenal	x	x	Fresh, green	Antimicrobial, antioxidant, cytotoxic
23	Hexyl acetate	x	x	Acid, citrus, fruit, green, herbal, rubber, spice, tobacco	Antimicrobial
28	6-Methyl 5-hepten-2-one	x	x	Citrus, fatty, green	Antimicrobial, insecticidal
29	1-Hexanol	x	x	Floral, sweet	Antifungal
32	Nonanal	x	x	Aldehydic, citrus, waxy	Antimicrobial, anticancer, cytotoxic
33	(*E*)-2-hexen-1-ol	x	x	Fresh, green, grass, leaf	Antimicrobial
41	2-Ethyl-1-hexanol	x	x	Citrus, fresh, floral, oil, sweet	Antimicrobial, cytotoxic
45	Benzaldehyde	x	x	Bitter almond	Antitumor, antioxidant, antimicrobial, cytotoxic
46	1-Octanol	x	x	Citrus, fatty, pungent	Antifungal
69	Benzyl alcohol	x	x	Blackberry, floral, fruit	Antimicrobial, antiparasitic
70	Phenylethyl alcohol	x	x	Floral, herbal, honey, pollen, rose, spice, sweet	Antimicrobial, antioxidant, antienzymatic
71	Benzonitrile	x	x	Bitter almond, sweet	Antimicrobial
76	Benzyl isothiocyanate	x	x	Cabbage, radish, vegetative	Anticancer, antibacterial, antifungal, anti-inflammatory, antioxidant

## Data Availability

All the data generated or analyzed during this study are included in this article.
